# Patterns and short term neurosurgical treatment outcomes of neonates with neural tube defects admitted to Felege Hiwot Specialized Hospital, Bahir Dar, Ethiopia

**DOI:** 10.1186/s12887-024-04837-5

**Published:** 2024-05-22

**Authors:** Yohannes Addisu, Gizachew Tadesse Wassie

**Affiliations:** 1 Department of Pediatrics and Child Health, School of Medicine, College of Medicine and Health Sciences, Bahir Dar University, Bahir Dar, Ethiopia; 2 Department of Epidemiology and Biostatistics, School of Public Health, College of Medicine and Health Sciences, Bahir Dar University, Po Box: 79, Bahir Dar, Ethiopia

**Keywords:** Pattern, Neural tube defects, Outcome, Neonates, Child health, Neural surgery, Ethiopia

## Abstract

**Background:**

Neural tube defects (NTDs) account for the largest proportion of congenital anomalies of the central nervous system and result from failure of the neural tube to close spontaneously between the 3rd and 4th weeks of in utero development. Prognosis and treatment outcome depends on the nature and the pattern of the defect. The nature of treatment outcomes and its pattern associated with grave prognosis is not well known in the study area.

**Objective:**

The aim of study was to determine the patterns and short term neurosurgical management outcomes of newborns with neural tube defects admitted at Felege Hiwot Specialized Hospital.

**Methods:**

Institutional based retrospective cross-sectional study among neonates, who were admitted at Felege Hiwot Specialized Hospital with neural tube defects from January 1^st^ to December, 30^th^, 2018 was conducted. All Charts of Neonates with confirmed diagnosis of neural tube defects were included as part of the study. Trained data collectors (medical interns) supervised by trained supervisors (general practitioners) collected the data using a pretested data extraction format. Data were coded, entered and analyzed using SPSS version 23 software. Frequency and cross tabulations were used to summarize descriptive statistics of data, and tables and graphs were used for data presentation.

**Result:**

About 109 patients had complete documentation and imaging confirmed neural tube defects. Myelomeningocele was the commonest pattern 70 (64.2%). Thoracolumbar spine was the commonest site of presentation 49(45%). The most common associated impairment was hydrocephalus 37(33.9%). Forty-five (41.1%) had multiple complications. The mortality rate was 7.3%, 44% were discharged with sequalae and 36.7% were discharged without impairment. The significant causes of death were infection 66.7% and Chiari crisis 33.3%.

**Conclusion:**

Myelomeningocele was the most frequent clinical pattern of neural tube defect and thoracolumbar spine was the commonest site. Isolated neural tube defect was the commonest finding. There were multiple complications after surgery accompanied with meningitis and hydrocephalus. The mortality rate among neonates with neural tube defects was considerably high. The commonest causes of death were infection and Chiari crisis.

## Background

Neural tube defects (NTDs) accounted for the largest proportion of congenital anomalies of the central nervous system (CNS) and resulted from failure of the neural tube to close spontaneously between the 3rd and 4th week of in utero development. The major NTDs include spina bifida occulta, meningocele, myelomeningocele, encephalocele, anencephaly, caudal regression syndrome, dermal sinus, tethered cord, syringomyelia, diastematomyelia, and lipoma involving the conus medullaris and/or filum terminale and the rare condition anencephaly. An estimated 240, 000 newborns die worldwide within 28 days of birth every year due to congenital disorders. Congenital disorders cause a further 170, 000 deaths of children between the ages of one month and five years with NTDs being one of the most serious and most common of these anomalies [1].

NTDs have a global prevalence ranging from 1 to 10 per 1000 live births [2], resulting in 300,000 to 400,000 infants born with conditions such as spina bifida and anencephaly each year. Despite being a significant cause of perinatal morbidity and mortality, NTDs are preventable. Anencephalic children typically do not survive beyond birth, while those with meningocele and myelomeningocele have higher survival rates due to medical and surgical intervention [2, 3].

NTDs are associated with substantial mortality, morbidity, disability, and psychological and economic costs in which an estimated 13% of neonatal deaths attributed to birth defects in low resource countries [3]. In Ethiopia, the prevalence of NTD reported as 10.9 per 1000 pregnancies in Debere Birhan referral hospital and 4.2/1000 in Addis Ababa [4]. Many are preventable with folic acid, and accesses to appropriate services that can result in improve survival and quality of life. Improved surveillance of all adverse outcomes is needed to improve the robustness of total NTD prevalence estimation, evaluate effectiveness of prevention through folic acid fortification, and improve outcomes through care and rehabilitation [4].

To effectively manage and supervise a child and their family dealing with NTD, a multidisciplinary team approach is necessary [2]. This team should include surgeons, physicians, and therapists, with one individual acting as the advocate and coordinator of the treatment program. The focus should be on optimizing functional outcomes for these children through coordinated multidisciplinary care from childhood and beyond. It is crucial to have an understanding of the current management of these conditions and the potential long-term prognosis to assist parents in deciding whether to proceed with the pregnancy or not. It is important to note that this study solely addresses 'open' neural tube defects [5].

Many infants with myelomengocele, meningiocele, encephalocele now survive, usually as a result of extensive medical and surgical care. The risk of early death among infants with open spina bifida varies considerably worldwide, depending not only on the severity of the lesion but also on such factors as the availability, use, and acceptance of medical and surgical treatment [6, 7]. Anatomical location of the spina bifida cases was cervical 29%, thoracic 28% and lumbar 43% regions, respectively [8, 9].

With improved neonatal and neurosurgical care the early mortality associated with neural tube defects has reduced and, the majority of children born with these defects survived in best setups [8]. But there is scarcity of data on burden of NTDs and its treatment outcome in lower-income countries. Local data are important to understand the real burden of the problem [1].

Based on our observations there are many patients with NTDs and neuro surgical procedures for their closure. But there was no study done on neurosurgical treatment outcome of NTDS previously in Bahir Dar and nationwide. Hence, this study aimed to describe the patterns of and treatment outcomes of infants with NTDs.

## Methods and materials

### Study area, period and design

The study was conducted at Felege Hiwot Specialized Hospital (FHSH), Amhara region, Ethiopia. A hospital based cross-sectional study was conducted in FHSH from January 1^st^ to 30^th^,2018.

### Study participants

Neonates with NTD and admitted in FHSH neonatal intensive care unit (NICU) from January 1^st^ to December 30^th^, 2018 were included. Out of the 136 neonates admitted to the NICU during this time, only 109 had complete chart documentation and were therefore included in the analysis. The remaining 27 newborns with NTDs with incomplete documentation were excluded.

### Data collection tools and procedures

Patients’ data were collected using semi-structured data extraction checklist from patient charts. Patterns of NTDs, the nature and site of the defect relative to spinal vertebrae and cranium, and short-term outcomes after treatment at discharge (including death, complications, or improvement) were collected. Short-term outcomes after treatment were determined within six months. The patient card number was obtained from the health management information system (HMIS) logbook of the NICU ward, and patient cards were retrieved from the card room. The diagnosis of NTD was based on clinical and Para- clinical evaluation (as documented by doctors in the patient's case files) and compared with their ultrasound results. The patient's history, including antenatal history, was obtained from the folders, along with imaging reports.

### Data processing and analysis

The filled data check lists were checked for completeness, and cleaned manually. Then, coded and entered into SPSS version.23 software, for analysis. Any logical and consistency error identified during data entry was corrected by revision of the original completed check lists. The data was analyzed after ensuring proper entry and subsequent cleaning. Frequency and cross tabulations were used to summarize descriptive statistics of data. Data were presented using text, tables and graphs.

## Results

### Socio demographic characteristics of participants

A total of 136 neonates were clinically diagnosed with NTDs. Of these, 109 patients had complete documentation and imaging confirmed neural tube defects result. The age distribution of studied neonates ranged from one day to 15 days and the mean age at presentation was two days. Sixty seven (61.5%) of newborns were males. Sixty (55%) of the cases were referral cases while forty-nine (45%) were born in FHSH..

### Patterns of presentation, associated impairment s and neurologic status of neural tube defects before surgery

Visible sac on the back was the commonest presentation seen in 47% of all NTD cases. It was followed by visible sac with hydrocephalus and paraparsis/plegia (22.9%) and visible sac with hydrocephalus only (21.1%). Nearly all neonates with neural tube defects 107 (98.2%) had documented imaging results.

Myelomeningocele was the commonest NTD, accounted for 70(64.2%) cases, followed by meningiocele and encephalocele accounted for 26(23.9%) and 13(11.9%) cases respectively. Thoracolumbar spine was the commonest site of presentation which accounts 49(45%), followed by lumbar area 38(34.9%). Fifty one, (46.8%) of neonates had isolated neural tube defects. The most common associated impairment was hydrocephalus, 37 (33.9%) cases followed by multiple congenital malformations, 12 (11%) cases. The most common neurologic finding associated with NTD was paraplegia 19(17.4%) followed by paraparsis 13(14.7%), the remaining 73(67%) had no neurologic finding (Table 1).

**Table 1:** Patterns of presentation, associated impairment s and neurologic status of neural tube defects before surgery over year in Bahir Dar, Ethiopia Felege Hiwot Specialized Hospital (*N* = 109)

Variables	Patterns	Frequency	Percent
Type of NTD	Encephalocele	13	11.9
	Meningiocele	26	23.9
	Myelomeningocele	70	64.2
Site of NTD	sacral	1	0.9
	Lumbosacral	5	4.6
	Lumbar	38	34.9
	Thoracolumbar	49	45
	thoracic	5	4.6
	Occipital	11	10.1
Associated impairment with neural tube defects before surgery	No impairment	51	46.8
	Hydrocephalus	37	33.9
	Club foot	9	8.3
	Multiple congenital malformations	12	11
Neurologic status before surgery	Paraplegia	19	17.4
	Paraparsis	16	14.7
	Normal	73	67
	Other	1	0.9

### Type of neurosurgical management done and complications after management

There are two types of neurosurgical procedures done at FHSH. Surgical repair of the defect was the leading procedure done in FHSH, accounted for 95(87.2%) cases and both surgical repair of the defect and shunt for hydrocephalus accounted for 14(12.8%). cases.

**Fig. 1:**
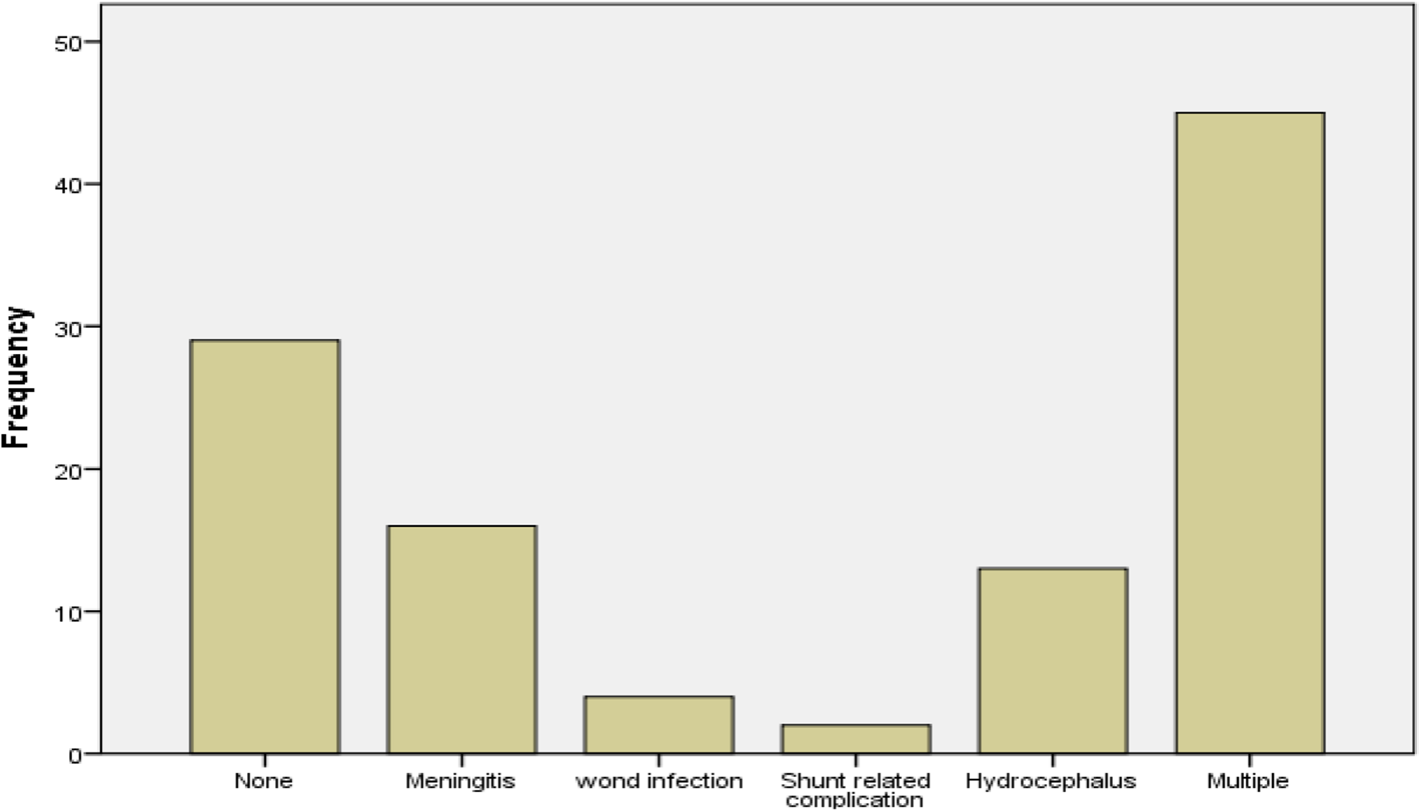
Complications after neurosurgical procedure among neonates with NTD in FHSH, Bahir Dar, Ethiopia

The majority (73.4%) of neonates had postoperative complications, while only 26.6% had no complications. The most common complications included multiple (two and more) complications (41.1%), meningitis (14.7%) and hydrocephalus (11.9%) as shown in (Fig. 1).

### Outcome status at discharge

Nine (7.3%) neonates with neural tube defects died, with infection (any infection following surgery in hospital before discharge) being the cause for 6 (66.7%) of deaths and Chiari crisis 3(33.3%). Meanwhile, 48(44%) were discharged with sequelae, including hydrocephalus and paralysis, and 40(36.7%) were discharged without impairment. Eight (11.9%) went against medical advice and their outcome was unknown. The mean duration of hospital stay was 22.4 days after admission.

## Discussion

Neural tube defects are the most common birth defect of the central nervous system and they occur at a range of 0.5–10 or more in 1,000 live births worldwide [10, 11]. It is resulting from incomplete closure of the neural tube during the first month of pregnancy [12, 13] associated with significant clinical complications that can affect survival and the quality of life for affected neonates and the family.

In this study, from 136 patients clinically diagnosed to have NTDs, only 109 patients had complete documentation and imaging confirmed neural tube defects. Sixty-seven (61.5%) of them were males. This figure is almost close comparable with studies done in Cameroon [2], Rowel Pindi [14] and New Delhi [15] which are male predominance. But studies done in Northern Saudi Arabia 57.1% were females and 42.9% were males [16]. This shows that female predominance. This predominance appears to be influenced by the presence of additional birth defects, geographical areas and other factors. And other potential explanations given for the predominance differences between male and female are in embryonic developments’ susceptibility to teratogenic insult.

Visible sac on the back was the commonest presentation of all NTD patients among NTD cases. It was followed by visible sac with hydrocephalus and paraparsis/plegia. This study agreed with study done in India, a visible sac over the back was the commonest presentation (72%) and a minority presented with neurological deficits [7].

In this study, myelomeningocele was the commonest pattern of NTDs, accounting 70(64.2%), which is consistent with studies done in India [7], Northern China [17], Turk [18], Zanzibar [19], Nigeria [20], Cameroon [2] and Black Lion Hospital in Ethiopia [8]. However, studies done in Saudi Arabia show that spina bifida occulta was the most common, accounting 57.1% followed by myelomeningocele at 28.6% [16]. Additionally, a study in New Delhi reported myelomeningocele at 50% 5 and meningocele at 50% [15]. and, contradicting the findings of this study. These variations may be explained by the influence of racial, geographical, nutritional, socioeconomic, and biological differences. This study shows that thoracolumbar spine was the commonest site of NTD accounting 49(45%), followed by lumbar area 38(34.9%) which is different from literatures like Saudi Arabia commonest site was lumbosacral area followed by lumbar area [16], In Pakistan [21] and sub Saharan Zambia [22] the commonest site was lumbar area and New Delhi [15], Turk [18] and Nigeria [20] the commonest site was lumbosacral area. The possible reason may be impaired progression of closure, and consequently the presence of a persistently open posterior neuropore, results in spina bifida, and the size of the ensuing lesion relates directly to the axial level at which closure stops.

Fifty-one (46.8%) of neonates were having isolated neural tube defects. The most common associated impairment was hydrocephalus 37(33.9%). This result agrees with studies done in India New Delhi [15], but NTDs with hydrocephalus was the commonest finding followed by isolated NTDs in studies done Cameroon [2], Pakistan [21], Turk [18] and Nigeria [20]. The possible explanation is related to the type of underlying etiology resulted NTDs.

The most common neurologic finding associated with NTD was paraplegia 19(17.4%) followed by paraparsis 13(14.7%), the remaining 73(67%) were not having neurologic findings.

Only surgical repair of the defect was the leading procedure done in FHSH accounting 95(87.2%) as compared to studies done in Serbia [12], India [7], China [15], and Turk [18] both repaired and ventriculo-peritoneal shunt are being done. The possible reason may be due to different socioeconomic status of the settings.

Twenty-nine (26.6%) neonates with NTDs had no complications after surgery. The remaining had complications after surgery, with forty five (41.1%) had multiple complications (two or more) which suggests that neural tube defects can be complex and challenging to treat, requiring careful post-operative management. Meningitis and hydrocephalus accounted 16(14.7%) and 13(11.9%) cases respectively. This finding was consistent with previous studies done in Zanzibar [19], while studies done in India [7] and Nigeria [20] showed a highest number of patients without complication after surgery. Ninety and seventy percent of procedures were without complications in India and Nigeria respectively. The possible explanation may be related to better health care set up in these countries.

Among 109 neonates with neural tube defects, 9(7.3%) were died, 48(44%) were discharged with sequealae, and 40(36.7%) were discharged without impairment. The most important causes of death were infection in 6(66.7%) cases and Chiari crisis in 3(33.3%) cases. Mortality rate of this study is higher than studies from Zanzibar [19] and Herzegovina [23]. One possible reason may be related to longer hospital stay that predispose for infection. Infection was the leading cause of death which is similar with mentioned literatures. Postoperative mortality rate in Saudi Arabia [16], New Delhi [15] and Uganda [24, 25] were found to be higher than this study. The possible explanation may be they have relatively longer period of follow up after discharge and the presence of complications before surgery significantly contributed to this high and the outcome was not known for neonates went against medical advice in this study.

## Conclusions

Myelomeningocele was the most frequent clinical pattern of neural tube defect and thoracolumbar spine was the commonest site. Additionally, isolated NTD was the commonest finding followed by NTDS with hydrocephalus. Moreover, there were multiple complications (two and above) after surgery, with meningitis and hydrocephalus being common.

The mortality rate among neonates with NTD was considerably high. The primary causes of death were infection and Chiari crisis. Therefore, further longitudinal research should be done to identify effective surgical interventions to manage neural tube defects.

## Data Availability

The datasets used and/or analyzed during the current study are available from the corresponding author on reasonable request.
